# Comparison Analysis between the Medication Efficacy of the Milnacipran and Functional Connectivity of Neural Networks in Fibromyalgia Patients

**DOI:** 10.3390/brainsci10050295

**Published:** 2020-05-15

**Authors:** Seong-Ho Kim, Min-Woo Lee, Min-Jae Kang, Sung Gun Lee, Jung-Goo Lee, Chi-Woong Mun

**Affiliations:** 1 Department of Internal Medicine, Inje University Haeundae Paik Hospital, Busan 48108, Korea; junjan@paik.ac.kr (S.-H.K.); sglee.ac@gmail.com (S.G.L.); 2 School of BME and u-HARC, Inje University, Gimhae, Gyeongnam 50834, Korea; Leemw1125@gmail.com (M.-W.L.); alswo88@dgmif.re.kr (M.-J.K.); 3 Neuroscience Research Institute, Gachon University, Namdong-daero 774beongil, Namdong-gu, Incheon 21565, Korea; 4 Medical Device Development Center, Daegu-Gyeongbuk Medical Innovation Foundation, 88 Dongnae-ro, Daegu 41061, Korea; 5 Department of Psychiatry, Inje University Haeundae Paik Hospital, Busan 48108, Korea; iybihwc@naver.com; 6 Paik Institute for Clinical Research, Inje University, Busan 47392, Korea; 7 Department of Health Science and Technology, Graduate School, Inje University, Busan 47392, Korea

**Keywords:** fibromyalgia, milnacipran, drug efficacy, graph theory, rs-fMRI, neural network connectivity

## Abstract

Milnacipran is a reuptake inhibitor of both serotonin and noradrenaline, used in the treatment of fibromyalgia with severe depression. However, few studies have been conducted on the efficacies of milnacipran drug on the functional connectivity of the neural network. The authors aimed to find the correlation between the drug efficacy and the changes in neural network in fibromyalgia patients. Resting-state-functional magnetic resonance imaging (rs-fMRI) were obtained before and after milnacipran drug administration. Graph theory indexes and small-worldness were calculated using preprocessed blood-oxygen-level-dependent signals from the rs-fMRI scans of 14 brain regions-of-interest. Statistical analyses were conducted to compare the topological network parameters. Significant changes in the neural network indexes appeared in three of the 14 brain regions-of-interest. In the pain network, the average path length on the left side of Brodmann area 32 was shortened. In the default mode network, functional connectivity changes were observed in the left lateral parietal cortex and medial prefrontal cortex. In the left lateral parietal cortex, the degree and betweenness centrality increased, whereas the clustering coefficient decreased. In the medial prefrontal cortex, local efficiency decreased. The small-worldness declined after milnacipran medication. The present results demonstrate that functional connectivity indexes in the brains of female fibromyalgia patients obtained from rs-fMRI data can be used as potential prognosis markers of milnacipran drug treatment.

## 1. Introduction

This research group mainly prescribes milnacipran (MLN) [1] and duloxetine [2] of serotonin-norepinephrine reuptake inhibitor (SNRI) medication for fibromyalgia (FM) patients with severe depression, and pregabalin and gabapentin of anti-seizure drug group for patients with sleep disorders or pain-sensitive reactions [2,3]. Occasionally, both medications may be prescribed when these three symptoms appear in combination. Although it is well known that the alleviating effects of these medications are more effective than other treatments, few studies have evaluated the efficacies of these drugs on human health, especially the nervous system. Authors have become interested in objective prognosis markers of the efficacy of MLN drug therapy in FM patients. MLN is a non-tricyclic compound that functions as a selective reuptake inhibitor of both serotonin and noradrenaline with a superior side effect profile. Gendreau et al. [1] reported that MLN improves the overall status of a patient in terms of global well-being, fatigue, pain, and a variety of symptom domains in the absence of serious adverse events.

The human brain can be modeled as a network in which the nodes in large-scale brain networks represent brain regions-of-interest (BROIs), while edges or links represent anatomical, functional, or effective connections between them [4,5]. The topological connectivity of the neural network related to the blood-oxygen-level-dependent (BOLD) signal can be quantitatively represented using graph theory, which can be used to intensively analyze the characteristics of functional connections within a brain region [6]. Thus, graph-based brain network analysis provides a theoretical framework associated with the topologies of structural and functional connectivity and can reveal novel insights regarding brain networks [4,5,7,8,9,10]. Rubinov et al. [4] reported that anatomical connections typically correspond to white matter tracts between pairs of BROIs, whereas functional associations correspond to temporal correlations in spontaneous brain activity, which may occur between pairs of anatomically unconnected regions. Therefore, brain network analysis may be a reliable and useful method for quantitative measures of structural–functional relationships that can be used to diagnose abnormal connectivity in patients with neurological and psychiatric disorders.

Analyses of rs-fMRI data by several research groups have shown that intrinsic neural connectivity within multiple neural networks is associated with spontaneous clinical pain in FM patients [3,11,12,13]. These research groups also reported that brain activity is higher in pain network areas but lower in the default mode network (DMN) in FM patients compared to normal subjects. The DMN is a network that contains constellation brain regions that are intrinsically deactivated by certain external stimuli and that are more active in the dormant state [1,8,13,14,15]. Moreover, it is known that pain affects one’s cognitive capacity and various DMN regions [13,16].

Based on these findings, the present study employed graph theory analyses of rs-fMRI data to address where and how longitudinal brain network profiles change in FM patients following treatment with MLN. The goal of this study was to identify prognosis indicators of MLN drug therapy by monitoring changes in the indexes of graph-based functional connectivity within the DMN and the pain network in the brains of FM patients who exhibited improved symptoms after treatment with MLN.

## 2. Materials and Methods

In this study, 32 FM patients diagnosed according to the 2010 fibromyalgia diagnostic criteria [17] were involved. The age-matched patients were selected after evaluating the following clinical tests: Fibromyalgia Impact Questionnaire (FIQ), Brief Fatigue Inventory (BFI), Beck Depression Inventory (BDI), Widespread Pain Index (WPI), Symptom Severity Scale Score (SSS), and the State-Trait Anxiety Inventory (STAI) and STAI-2 scales. A total of 10 female FM patients who responded to MLN were enrolled. Following their diagnosis of FM, the patients underwent routine clinical treatment with MLN as prescribed by medical specialists. The criterion for a positive response was a >50% reduction in visual analog scale scores after taking the medication. Of the 10 female patients who responded to the MLN drugs, two were excluded due to severe motion artifacts in the MR images. Therefore, eight patients with a mean (± standard deviation) age of 51.15 ± 5.28 years (range: 40–56 years) were included in the final analyses.

The MR images were obtained using a 3.0-Tesla MRI scanner (Philips; Achieva, Netherlands). All rs-fMRI data were acquired using multi-slice EPI with the following parameters: TR/TE = 3000/30 ms, flip angle = 65°, matrix size = 220 × 220, field of view (FOV) = 128 mm, slice thickness = 4 mm, and scan time = 7 min 30 s. Anatomical images were obtained from three-dimensional T1-weighted MRI scans using a turbo field echo sequence with the following parameters: TR/TE = 9.9/4.6 ms, flip angle = 8°, matrix size = 240 × 240, FOV = 240 mm, and slice thickness = 1 mm.

The obtained rs-fMRI data were preprocessed using Statistical Parametric Mapping 8 (SPM 8; Welcome Trust Centre for Neuroimaging, London, UK) software [18] based on MATLAB^®^ 2015b (The MathWorks Inc.; Natick, MA, USA). Slice realignments of the rs-fMRI images were performed to correct fine motions of the head during the MRI scan, and the slice timing was corrected to reduce the time-delay error associated with multi-slice rs-fMRI scans. Co-registration between the rs-fMRI and anatomical images was conducted to compensate for the insufficient spatial resolution of rs-fMRI. After segmentation of the anatomical images, spatial normalization was performed to match the image volumes of the functional and anatomical images to the template volume images using 152 Montreal Neurological Institute (MNI) template images as a standard space model. Finally, a smoothing process was performed using a 6-mm full width at half maximum isotropic Gaussian kernel to improve the signal-to-noise ratio. Functional connectivity in the neural networks was analyzed in the following 14 brain areas: bilateral Brodmann areas 1–3, Brodmann areas 13, Brodmann areas 32, the medial prefrontal cortex (mPFC), the posterior cingulate cortex (PCC), and the left and right lateral parietal cortices. These BROIs can be classified into the pain network (bilateral regions of Brodmann areas 1–3, Brodmann areas 13, and Brodmann areas 32), and the DMN (mPFC, PCC, left lateral parietal cortex, and right lateral parietal cortex).

Using the Functional Connectivity Toolbox (CONN17e: Whitfield-Gabrieli and Nieto Castanon; MIT, MA, USA) [19], various algebraic brain connectivity indexes were obtained with the adjacency matrix (14 × 14) constructed for the 14 BROIs in each of the eight patients. The BROIs were intended to cover the four areas that correspond to the DMN and the 10 areas that correspond to the pain network. Analyses of the graph theory indexes were derived from the adjacency matrix. The results from each graph theory index as a marker of drug efficacy were presented using the NeuroMArVL (http://immersive.erc.monash.edu.au/neuromarvl) package [20]. In the analysis of functional integration of the entire brain, the range of connection density was varied (i.e., sparsity ranged from 5% to 40% to allow for a proper estimation of small-world parameters while minimizing the number of spurious edges in each network) [21,22]. In this process, the clustering coefficient and characteristic path length of the real networks and random networks were calculated at every 5% increment of connection density *k*. A random network was generated by randomly rewiring each of the edges 1000 times with connection density *k*. After calculating the fractions g(*k*) of the clustering coefficients and the fractions (*k*) of the characteristic path lengths between the measured real-world networks and generated random networks at each connection density *k*, small-worldness s(*k*) was obtained using the following definition: s(*k*) = g(*k*)/l(*k*).

Non-parametric statistical tests were conducted using the statistical computing and graphics software R (version 3.61 for Windows, Vienna, Austria). The brain connectivity indexes resulting from the graphical analysis of the rs-fMRI BOLD signals obtained before and after MLN treatment were compared using Wilcoxon signed-rank tests.

All patients involved in this study agreed to informed written consent in accordance with the Institutional Review Board at Inje University Haeundae Paik Hospital (No. 2012-058).

## 3. Results

**Figure 1:**
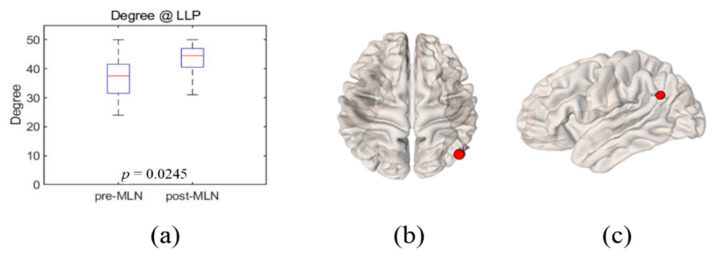
Illustrations of the degree of change in functional connectivity of neural network at the left lateral parietal cortex in fibromyalgia (FM) patients due to milnacipran (MLN) drug therapy. (**a**) Boxplots (left: before medication, right: after medication) of the degree distribution at the left lateral parietal cortex. This position is indicated by red circular markers on the 3D brain surface in (**b**) transverse and (**c**) lateral views. Red color markers imply that the degree index statistically increased due to drug therapy.

**Figure 2:**
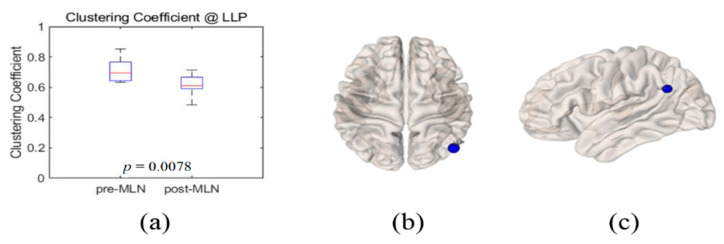
Illustrations of clustering coefficient change at left lateral parietal cortex as the functional connectivity of neural network in fibromyalgia patients’ brains due to Milnacipran drug therapy. (**a**) Boxplots (left: before medication, right: after medication) of the distribution of the clustering coefficient at left lateral parietal cortex. This position is indicated by blue-colored small disk on 3D brain surface in (**b**) transverse and (**c**) lateral views. Blue color markers imply that the clustering coefficient statistically decreased due to drug therapy.

**Figure 3:**
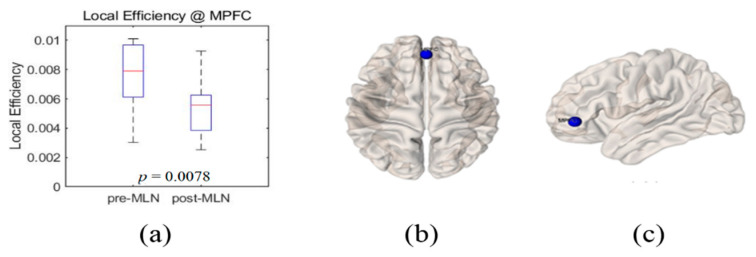
Illustrations of local efficiency change at medial prefrontal cortex (mPFC) as the functional connectivity of neural networks in fibromyalgia patients’ brains due to Milnacipran drug therapy. (**a**) Boxplots (left: before medication, right: after medication) of local-efficiency-index distributions at mPFC site. Blue markers indicate this position on the 3D brain surface in (**b**) transverse and (**c**) lateral views. The blue color of the markers implies that the local-efficiency index statistically decreased due to drug therapy.

**Figure 4:**
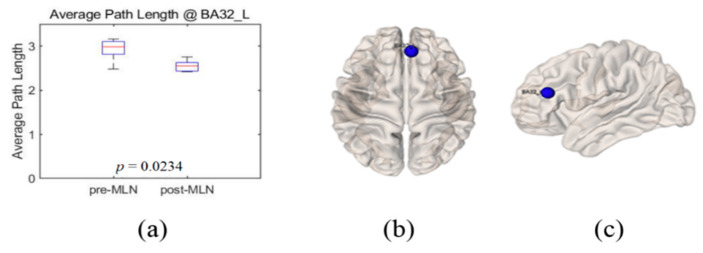
Illustrations of average path length change at left Brodmann areas 32_L as the functional connectivity of neural network in fibromyalgia patients’ brains due to Milnacipran drug therapy. (**a**) Boxplots (left: before medication, right: after medication) of the distribution of average path length at left Brodmann areas 32. Blue-colored markers indicate this location on the 3D brain surface in (**b**) transverse and (**c**) lateral views. Blue color markers imply that the average-path-length index statistically decreased due to drug therapy.

**Figure 5:**
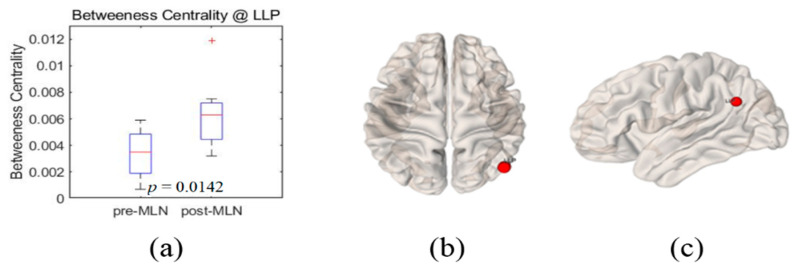
Illustrations of betweenness centrality change at the left lateral parietal cortex as the functional connectivity of neural network in fibromyalgia patients’ brains due to Milnacipran drug therapy. (**a**) Boxplots (left: before medication, right: after medication) of the distribution of the betweenness-centrality index at left lateral parietal cortex. The red markers indicate this location on the 3D brain surface in (**b**) transverse and (**c**) lateral views. Red color markers imply that the betweenness-centrality index statistically increased after the treatment of the MLN medication.

Significant changes (*p* < 0.05) in the functional connectivity indexes of the neural networks in treating FM patients with MLN medication are shown in Figure 1, Figure 2, Figure 3, Figure 4 and Figure 5 as follows: the degree and clustering coefficients at left lateral parietal cortex (Figure 1 and Figure 2, respectively), local efficiency at mPFC (Figure 3), average path length at the left Brodmann areas 32 (Figure 4), and betweenness at left lateral parietal cortex (Figure 5). Global efficiency was not presented here due to the lack of significant changes following MLN treatment. A pair of boxplots in the (a) panel of each figure shows the distributions of the corresponding neural network connectivity indexes before (left boxplot) and after (right boxplot) MLN treatment. The corresponding locations are indicated by circular markers on the top and lateral views of the cortical surfaces in the (b) and (c) panels, respectively. The red circular marker indicates an increase in the index distributions due to drug therapy, while the blue indicates a decrease in the index distributions. These activated or deactivated regions were represented at the title of each boxplot (a) and also indicated by small red and blue discs at the top (b) and side (c) view of brain surface pictures. A small disc color of red indicates an increase in brain connectivity index after MLN drug treatment and a decrease in blue.

### 3.1. Degree

The degree of each individual node illustrates an important marker of network development and resilience [4,8], and the corresponding nodes interact structurally or functionally with many other network regions. Figure 1a shows the distributions of the degree index, which increased with significant level *p* = 0.03 at left lateral parietal cortex in DMN after MLN drug therapy. The red circular markers in Figure 1b,c indicate that the degree index values significantly increased at the left lateral parietal cortex site due to drug treatment; there were no significant results in other BROIs.

### 3.2. Clustering Coefficient

The boxplots in Figure 2a illustrate the index distribution measures of the clustering coefficient at the left lateral parietal cortex in the DMN before (left boxplot) and after (right boxplot) MLN medication. The blue circular markers in Figure 2b,c indicate that there was a significant decrease in the index values of the clustering coefficient at the left lateral parietal cortex (*p* = 0.01) after MLN drug therapy. There were no significant statistical changes in the clustering coefficient index at the other BROIs.

### 3.3. Local Efficiency

Decreased index distribution of the local efficiency with significant level *p* = 0.01 at mPFC of the FM patient’s brain were depicted by box plots in Figure 3a before (left box plot) and after (right box plot) the medication. This corresponding site is shown by small blue disks in Figure 3b,c on the 3D brain model in axial (left) and sagittal (right) directions. The local efficiency indexes at the other 13 BROIs did not show statistical changes in significant level (*p* > 0.05).

### 3.4. Average Path Length

The index distributions of average path length at the Brodmann areas 32_L site were represented by box plots in Figure 4a before (left box plot) and after (right box plot) the treatment of MLN medication. The corresponding site was indicated by blue markers on the 3D brain surfaces in Figure 4b,c. The average path length at the left Brodmann areas 32 region shortened with a significant level (*p* = 0.02) after MLN treatment. Looking at the average path length values in the remaining BROIs, they had a tendency to decrease or to maintain similar values although they are not statistically significant (*p* > 0.05).

### 3.5. Betweenness Centrality

Index increase of the betweenness centrality (Hubness) after MLN medication occurred at the left lateral parietal cortex with significant level *p* = 0.01 and boxplots shown in Figure 5a before (left boxplot) and after (right boxplot) MLN medication depict their distribution changes. Small red-color markers on the 3D brain model in Figure 5b,c represent this left lateral parietal cortex site. The index values of the betweenness centrality in all other BROIs of the DMN and pain network tend to increase even they were not statistically significant (*p* > 0.05).

### 3.6. Global Efficiency

There were no significant changes in the global efficiency indices in both the DMN and the pain network after the MLN medication. However, global efficiency showed a tendency to increase in most pain network areas, except right Brodmann areas 1, and in most default mode network areas, except mPFC, even though they were not statistically significant (*p* > 0.05) in all BROIs.

### 3.7. Small-Worldness

**Figure 6:**
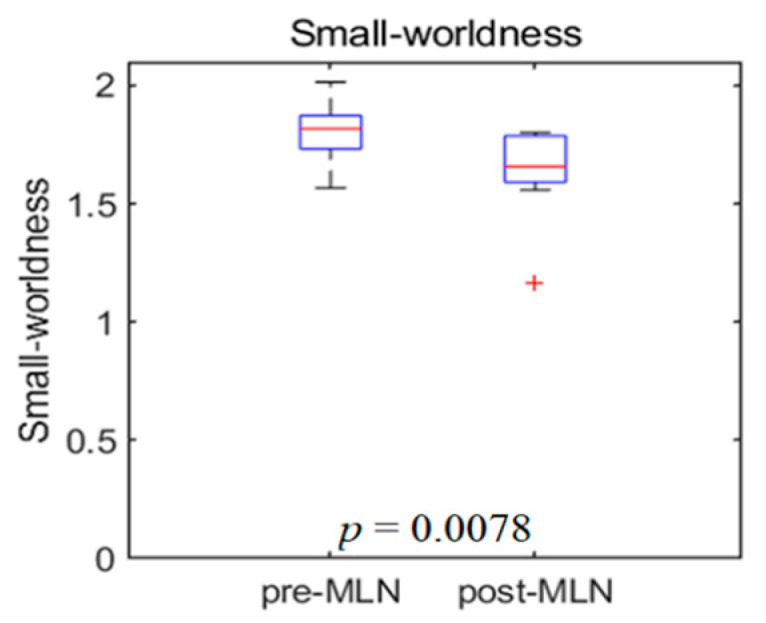
Boxplot illustrates the distribution change of the small-world as the functional connectivity of neural networks in fibromyalgia patients’ brains due to MLN drug therapy. Left: before medication, right: after medication.

Figure 6 shows the boxplots of the measures of small-worldness in patients with FM. The left and right boxplots show the results before and after MLN drug treatment, respectively. There was a significant difference of small-worldness between before and after MLN medication (*p* = 0.01) at 25% of connection density.

## 4. Discussion and Conclusions

The goal of this study was to identify prognosis markers of MLN efficacy in FM patients who responded to this drug by comparing resting-state functional connectivity in neural networks. The authors assessed seven indexes—degree, clustering coefficient, local-efficiency, average path length, betweenness centrality, and small-worldness—of graph-based functional connectivity in the DMN and the pain network was assessed, and six alterations in the neural networks were observed after MLN therapy (Figure 1, Figure 2, Figure 3, Figure 4, Figure 5 and Figure 6).

The degree is an indicator of the connectivity within the surrounding neural network area in the brain, and it has the meaning of an important marker of development and resilience [4,7,8,15,23]. From this, authors infer that the augmentation of the degree index in the left lateral parietal cortex area as shown in Figure 1 resulted from the restoration of the functional connectivity due to MLN drug treatment. The clustering coefficient in functional network implicates the prevalence of an organization of statistical dependencies of segregated neural processing [4]. As shown in Figure 2, the clustering coefficient decreased in the left lateral parietal cortex in the DMN region after MLN drug therapy, but no alterations were found in the pain network area. Therefore, it is presumed that MLN medication decreases the efficiency of information transmission in DMN; meanwhile, it does not affect the information transmission efficiency in the pain network region. The local efficiency, which measures the ability or the efficiency of information transmission within a given neural network site [4,6], declined in the mPFC area (Figure 3). This finding is consistent with the investigation of Napadow et al. [11], who reported that the mPFC is a cardinal node of the DMN and that the BOLD signal in the mPFC is correlated with spontaneous pain intensity. Taken together, these findings suggest that increased intrinsic DMN connectivity to the insular cortex is directly correlated with increasing levels of spontaneous clinical pain in FM patients. The average path length, the functional distance between all pairs of regions in a network, shortens with significant level at the left Brodmann areas 32 area (Figure 4). This finding seems to imply two facts. The first is that the pain network, including the insular cortex, has been localized, and the second is that the hyperactivity of the pain network has been inhibited after drug administration or relieving pain. The betweenness centrality, which has been implicated in the constitution of putative hubs that connect disparate parts of a network [4,8], significantly increased in the left lateral parietal cortex after MLN treatment (Figure 5). It seems that this phenomenon resulted from the restoration of efficient information integration in the DMN due to the antidepressant efficacy of MLN.

The default mode network plays a role in sustaining basic brain activity in the absence of external stimuli. Additionally, it also maintains a state of over-activation and will sensitively respond to noxious (hyperalgesia) and non-noxious (allodynia) stimuli [24]. Furthermore, the authors infer that the pain signals may be replaced by other signals. Wang et al. [6] insisted that the brain’s intrinsic activity is organized as a small-world network with highly connected hub regions. According to neuropsychological examinations, FM patients have higher anxiety and greater degrees of depressed feelings, fatigue, and pain sensitivity than healthy people. A comparison study of brain structural and functional network topologies between a control group and FM patients identified abnormal network connections in patients with neurological or psychiatric disorders [19]. Napadow et al. [12] reported that FM patients tend to exhibit higher neural connectivity in the DMN than healthy controls. MLN treatment is efficacious for both pain and depression, although its pain-relieving effects are greater in patients without depression [1]. Based on the present findings, it appears that MLN treatment increases the network connectivity of the DMN rather than decreasing internal connections within the pain network.

**Figure 7:**
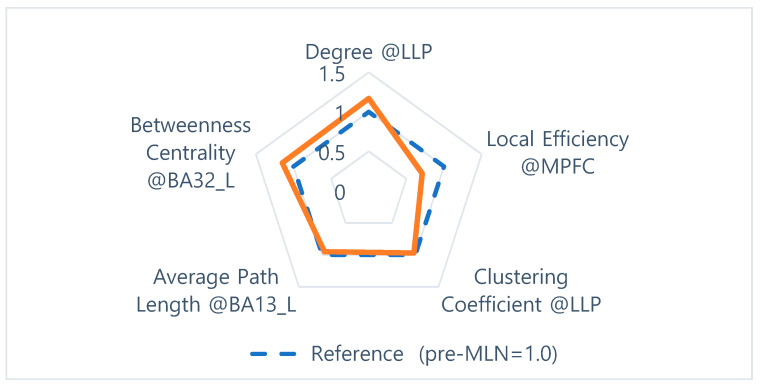
Multivariable comparison of the neural network indexes before and after MLN drug treatment (normalized by the index of pre-medication).

Napadow et al. [11] reported that functional connectivity in brain regions, excluding the insular cortex and secondary somatosensory cortex, from the DMN is higher in FM patients. To visually identify potential candidate markers of the effectiveness of MLN treatment among brain-connected indexes that showed significant differences after drug treatment, a two-dimensional multivariate graph, known as a Radar chart, was used (Figure 7). The findings revealed that the largest significant decreases occurred in local efficiency, whereas the degree and the betweenness centrality increased to a similar extent. Meanwhile, the average path length and clustering coefficient were relatively small in variation compared to the other indexes.

There are several limitations to this study. First, due to budgetary constraints, the number of patients included in this study was insufficient for robust statistical analysis. Regardless, the use of graph-based analyses of functional connectivity in neural networks in FM patients identified several possible markers of MLN treatment efficacy. In conclusion, this study demonstrated that graph-based analyses in combination with rs-fMRI could be used as a platform to explore predictive markers of MLN drug efficacy of FM patients by monitoring changes in functional connectivity within the topology of the neural network.
